# Frequent early cognitive impairment in both pediatric MS and MOGAD

**DOI:** 10.1177/13524585261465586

**Published:** 2026-08-06

**Authors:** Michiel Simon Jan Buijze, Sandy Molenaar, Rosa Verkerk, Maud Alida Quist, Willemijn Marije Carolien van der Vlugt, Rinze Frederik Neuteboom

**Affiliations:** Division of Pediatric Neurology, Department of Neurology, Erasmus MC University Medical Center Rotterdam, Rotterdam, The Netherlands; Division of Pediatric Neurology, Department of Neurology, Erasmus MC University Medical Center Rotterdam, Rotterdam, The Netherlands; Department of Pediatric Neuropsychology, Erasmus MC University Medical Center Rotterdam, Rotterdam, The Netherlands; Division of Pediatric Neurology, Department of Neurology, Erasmus MC University Medical Center Rotterdam, Rotterdam, The Netherlands; Department of Pediatric Neuropsychology, Erasmus MC University Medical Center Rotterdam, Rotterdam, The Netherlands; Division of Pediatric Neurology, Department of Neurology, Erasmus MC University Medical Center Rotterdam, Rotterdam, The Netherlands

**Keywords:** multiple sclerosis, myelin oligodendrocyte glycoprotein antibody-associated disease, cognitive impairment, cognitive profile, children

## Abstract

**Background:**

Studies on early cognitive impairment (CI) in pediatric multiple sclerosis (MS) are heterogeneous and data on pediatric myelin oligodendrocyte glycoprotein antibody-associated disease (MOGAD) are scarce.

**Objectives:**

To evaluate early cognitive dysfunction in both diseases and delineate disease-specific neurocognitive profiles.

**Methods:**

A cross-sectional study of children with MS and MOGAD assessed neurocognition within 2 years after disease onset using a comprehensive test battery. Test results were converted to *Z*-scores. CI was defined as *Z*-scores below −1.5 in ⩾2 domains. Children were compared by CI status and domain deficits quantified with composite *Z*-scores.

**Results:**

Sixty-seven participants were analyzed (MS *n* = 36, MOGAD *n* = 31). Despite normal full-scale intelligence quotient, 47% of MS children and 43% of MOGAD children (acute disseminated encephalomyelitis excluded) met criteria for CI. Domain-specific impairments were prominent in complex attention, with significantly reduced composite *Z*-scores (MS: −1.24, *p* < 0.001; MOGAD: −1.12, *p* = 0.019). MS children additionally showed lower scores on tasks of visuospatial processing and visual memory. Only fatigue was significantly associated with the presence of CI (PedsQL fatigue *Z*-score: −2.61 vs −0.75, *p* = 0.005).

**Conclusion:**

In both pediatric MS and MOGAD, early CI is prevalent with difficulties in complex attention. Visuospatial cognition appears to be an additional vulnerable area for children with MS.

## Introduction

Early recognition of cognitive impairment (CI) in children with acquired demyelinating syndromes (cADS), including pediatric multiple sclerosis (MS) and myelin oligodendrocyte glycoprotein antibody-associated disease (MOGAD), is crucial due to its potential impact on daily functioning, academic achievement, and long-term outcomes.^1,2^ Although approximately 30% of children with MS are thought to develop CI, significant variability in study designs, cognitive assessment methods, and timing of assessment complicate conclusions regarding specific cognitive deficits in the early disease course.^3,4^ Although emerging evidence in adult MOGAD suggests cognitive involvement,^5–7^ the extent and characteristics of CI in children with MOGAD remain largely unknown.^8^ Systematic neuropsychological assessment (NPA) is essential to obtain unbiased and reliable information on cognitive functioning in both diagnosis groups, thereby enabling meaningful evaluation of brain health and informing clinical monitoring and early intervention strategies. Therefore, our primary aim is to assess the presence of CI in the early disease course in children with MS and MOGAD using a comprehensive NPA encompassing all major cognitive domains. As secondary objectives, we will extensively characterize the neurocognitive profile, explore associated factors and explore the relationship between objective CI and self-reported cognitive complaints.

## Methods

### Study design

This is a cross-sectional analysis of neurocognitive data of children with cADS from an ongoing Dutch nationwide observational cohort study, the PROUDkids2.0 study (“Predicting Outcome and Disease Course in acquired demyelinating syndromes in Childhood”).^9^

### Study participants

Participants were consecutively recruited from medical centers throughout the Netherlands between 2018 and 2025. Referral for NPA was offered routinely to eligible participants who visited the study coordination center, which is a specialized pediatric neuroinflammatory outpatient clinic at the Erasmus University Medical Center. For this study, only children with MS and MOGAD were selected. MS was diagnosed according to the 2017 McDonald criteria^10^ and MOGAD according to the international MOGAD criteria.^11^ NPA was not offered to children ineligible for valid assessment because of young age, pre‑existing intellectual disability, recent NPA elsewhere or lost to follow‑up. Children who underwent NPA could be excluded from analysis based on predefined criteria, including concomitant non‑demyelinating central nervous system (CNS) disease, recent relapse or corticosteroid use, insufficient Dutch proficiency, or insufficient cognitive assessment (<2 cognitive domains tested).

### Data collection

Demographic and clinical data were collected using standardized forms. Demographic variables included age, sex, ethnicity, and migration background. Clinical data comprised the presenting clinical phenotype, relapsing disease course, Expanded Disability Status Scale (EDSS), self-reported cognitive complains within the first year after disease onset, and the use of immunomodulatory treatments (IMTs). In addition, first event brain magnetic resonance imaging (MRI) data and self-reported questionnaires assessing fatigue (Dutch version of the Pediatric Quality of Life Inventory Multidimensional Fatigue Scale (PedsQL Fatigue))^12^ and depression (Children’s Depression Inventory (CDI))^13^ were collected (Supplementary 1). Questionnaires were completed within 2–3 months prior to NPA.

### NPA

A standardized age-adapted NPA was employed by an experienced pediatric neuropsychologist. The test battery was designed to comprehensively evaluate the principal cognitive domains, defined by the Diagnostic and Statistical Manual of Mental Disorders, Fifth Edition (*DSM*-5): complex attention, executive function, learning and memory, perceptual-motor function and language.^14^ Supplementary 1 provides a definition overview regarding cognitive domains and subdomains. Assessments were conducted in Dutch using validated materials. Testing time did not exceed 3 hours per participant, with assessments occasionally split over 2 days when necessary. Tests were selected based on psychometric validity, age-appropriateness and clinical relevance.

In children aged 6 years and older, the following test battery was administered as demonstrated in Table 1. Tests for children with acute disseminated encephalomyelitis (ADEM) were administered whenever feasible based on age and task engagement.

**Table 1.** Neuropsychological assessment test battery.

Cognitive domain	Subdomain	Test	Measure	Abbreviation
Complex attention	Attention	Bourdon-Vos Test	Standard deviation score	BVT SD
		Stroop Color Word Test	Interference score	SCWT Interference
	Processing speed	Bourdon-Vos Test	Mean time score	BVT Time
		Wechsler Intelligence Scale for Children/Wechsler Adult Intelligence Scale	Processing speed index	PSI
Executive functioning	Planning/organization	Behavioral Assessment of the Dysexecutive Syndrome	Key search test score	BADS Key search
	Cognitive flexibility	Trail Making Test	Contrast score	TMT Contrast
	Working memory	Wechsler Intelligence Scale for Children/Wechsler Adult Intelligence Scale	Working memory index	WMI
Learning and memory	Verbal memory	15-Word Test	Immediate recall score	15-WT Immediate
			Delayed recall score	15-WT Delayed
	Visuospatial memory	Rey Complex Figure Test	Immediate recall score	RCFT Immediate
			Delayed recall score	RCFT Delayed
Visuospatial reasoning	Visuospatial cognition	Rey Complex Figure Test	Copy trial score	RCFT Copy
		Wechsler Intelligence Scale for Children/ Wechsler Adult Intelligence Scale	Visual Spatial Index^a^	VSI
		Beery–Buktenica Developmental Test	Visual motor integration score	Beery VMI
Language	Verbal abilities	Wechsler Intelligence Scale for Children/ Wechsler Adult Intelligence Scale	Verbal comprehension index	VCI

a To make a comparison between the WISC-V-NL and the WAIS-IV-NL possible, a composite score of the subtests Block Design and Visual Puzzles was calculated for the WAIS-IV-NL to match the VSI of the WISC-V-NL.

### Data analysis

All standardized scores were converted to *Z*-scores based on appropriate reference cohorts for direct cross-test comparison (Supplementary 1). All analyses were stratified in three diagnosis groups: MOGAD with ADEM phenotype (aMOGAD), MOGAD with non-ADEM phenotype (naMOGAD) and MS, because ADEM represents a biologically and clinically distinct entity and comprehensive NPA was frequently not feasible. CI was defined as mild (MCI) if test *Z*-scores fell below −1.5, and severe (SCI) if below −2.0, in two or more cognitive domains relative to normative data. This multidomain approach reduces false positives due to random variation or test noise and thereby increases specificity and clinical relevance.

Deviation from the normative mean was assessed using one‑sample Wilcoxon signed‑rank tests with a one‑sided alternative (median *Z* < 0), while differences between diagnosis groups were examined using two‑sample Wilcoxon rank‑sum tests. A polar plot was created to visually highlight the strong and weak test scores, linking them to cognitive subdomains, with median *Z*-scores and interquartile range (IQR) displayed for each subtest.

To further characterize the neurocognitive profiles, domain‑level composite *Z*‑scores were calculated as the mean of test *Z*‑scores and assessed for deviation from the normative mean using one‑sample Wilcoxon signed‑rank tests (one‑sided, median *Z* < 0). Domain-specific impairments (DSIs) were defined as mild (composite *Z*-score < −1.5) and severe (composite *Z*-score < −2.0). Observed proportions were compared with the expected proportions under a normal distribution (6.7% for *Z* < −1.5; 2.3% for *Z* < −2.0) using one‑sample binomial tests. Demographic, clinical, radiological and patient-reported variables were compared between participants with CI (defined as fulfilling criteria for MCI) and those without impairments for the MS and naMOGAD group using Wilcoxon rank‑sum test or chi-square/Fisher’s exact test, as appropriate. The concordance corrected for chance for the relationship between CI and patient or parent-reported cognitive complaints was calculated with Cohen’s kappa. Preliminary analysis revealed limited test availability for aMOGAD children, leading to exclusion of this group from most analyses. However, descriptive data for aMOGAD are presented for its valuable insights. Analyses were performed using Rstudio (version 4.4.1). The level of statistical significance was defined as 0.05. To account for multiple testing, the false discovery rate (FDR) was controlled using the Benjamini–Hochberg method.

### Standard protocol approvals, registrations and patient consents

The PROUD-kids 2.0 study was approved by the Erasmus Medical Centre Ethics Committee. Informed consent was obtained from all participants, with parental consent for those under 16 and additional assent from participants aged 12 and older.

## Results

### Study participants

Figure 1 shows the participant selection after applying exclusion criteria. In total, 67 participants were included in the analysis, consisting of 36 children with MS, 21 with naMOGAD and 10 with aMOGAD. Participant characteristics are demonstrated in Table 2. The relatively low number of children with aMOGAD reflects limited feasibility of NPA in younger children. The mean disease duration at NPA was similar and less than 1 year for all three diagnosis subgroups. Statistically significant differences were found for age at NPA, clinical phenotype, EDSS and both acute and maintenance IMT. More than 80% of children with MS used maintenance IMT, of whom almost 30% were treated with high-efficacy therapy (natalizumab, ocrelizumab or fingolimod). Among children with MOGAD, all but one received intravenous corticosteroids, with limited use of additional acute treatment, followed by oral corticosteroid tapering in only two children, and maintenance IMT in three children due to relapses.

**Figure 1.**
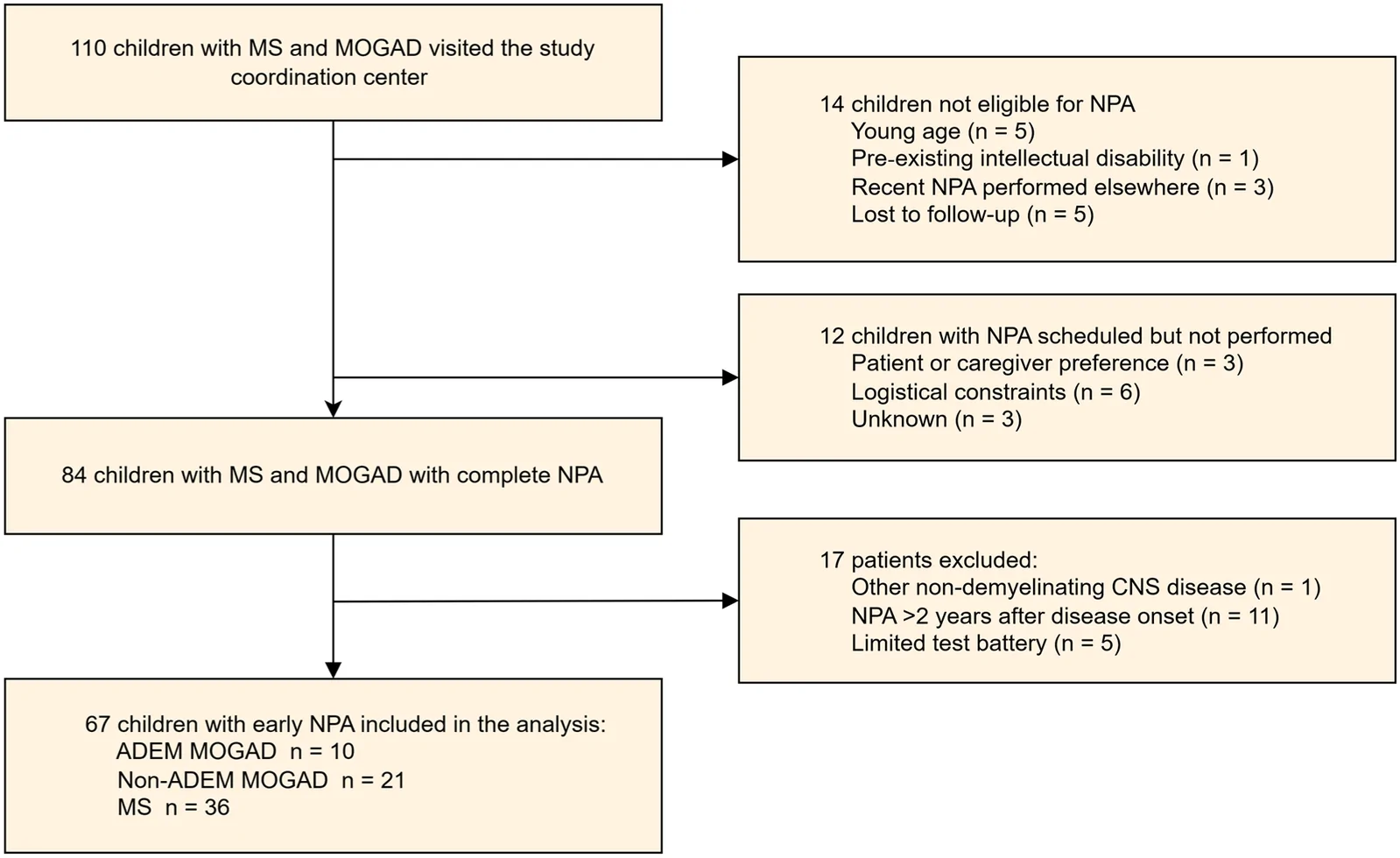
Flowchart of study participant selection.

**Table 2.** Characteristics of study cohort.

	ADEM MOGAD *N* = 10	Non-ADEM MOGAD *N* = 21	MS *N* = 36	*p*-value
**Demographical characteristics**				
**Age at time of NPA in years, mean (SD)**	6.3 (3.0)	12.9 (3.0)	15.3 (2.2)	<0.001*
**Female sex**, *n* **(%)**	8 (80%)	9 (43%)	26 (72%)	0.056
**Born in Netherlands**, *n* **(%)**				0.90
Yes	9 (90%)	18 (86%)	30 (83%)	
No	0	2 (9%)	4 (11%)	
Unknown	1 (10%)	1 (5%)	2 (5.6%)	
**European origin**, *n* **(%)**				0.70
Yes	5 (50%)	12 (57%)	17 (47%)	
No	4 (40%)	8 (38%)	18 (50%)	
Unknown	1 (10%)	1 (5%)	1 (2.8%)	
**Clinical characteristics**				
**Clinical phenotype**, *n* **(%)**				<0.001*
ADEM	10 (100%)	NA	0	
CIS hemispheral	NA	0	3 (8.3%)	
CIS infratentorial	NA	1 (5%)	11 (31%)	
ON	NA	8 (38%)	4 (11%)	
Other/polyfocal^a^	NA	5 (24%)	4 (11%)	
TM	NA	7 (33%)	14 (39%)	
**Disease duration at time of NPA in months, mean (SD)**	9.6 (4.6)	10.4 (3.8)	8.9 (3.5)	0.22
**Relapsing disease**, *n* **(%)**	2 (20%)	5 (24%)	13 (36%)	0.50
**EDSS at time of NPA, median (IQR)**	1.0 (0–2.0)	1.5 (1.0–2.0)	2.0 (1.5–2.5)	0.026*
**Acute treatment at first event**, *n* **(%)**	9 (90%)	21 (100%)	16 (44%)	<0.001*
Intravenous corticosteroids^b^	9 (100%)	21 (100%)	16 (100%)	
Intravenous immunoglobulin^b^	3 (33%)	2 (9.5%)	1 (6.3%)	
Plasma exchange therapy^b^	0	2 (9.5%)	0	
**Oral corticosteroid taper at first event**, *n* **(%)**	0	2 (9.5%)	3 (8.3%)	1.0
**IMT maintenance treatment**, *n* **(%)**	2 (20%)	3 (14%)	30 (83%)	<0.001*
Moderate efficacy therapy^c^	NA	NA	22 (73%)	
Dimethyl fumarate			3 (10%)	
Glatiramer acetate			2 (6.7%)	
Interferon beta			15 (50%)	
Peginterferon beta			1 (3.3%)	
Teriflunomide			1 (3.3%)	
High efficacy therapy^c^	NA	NA	8 (27%)	
Fingolimod			1 (3.3%)	
Natalizumab			6 (20%)	
Ocrelizumab			1 (3.3%)	
Other^c^				
Intravenous immunoglobulin	2 (100%)	2 (67%)	0	
Rituximab	0	1 (33%)	0	
**Test battery characteristics**				
**Number of tests, median (IQR)**	3.5 (3–8.25)	11 (10–13)	11 (10–12.2)	<0.001*
**Number of domains tested, median (IQR)**	3 (3–5)	5 (5–5)	5 (5–5)	<0.001*
**Patient-reported outcomes**				
**Fatigue scale *Z*-score (child), median (IQR)** Missing, *n*	ND	−0.91 (−1.79, 0.29) 6	−1.24 (−2.69, 0.07) 6	0.29
**Fatigue scale *Z*-score (parent), median (IQR)** Missing, *n*	−0.93 (−1.89, −0.13) 4	0.11 (−1.20, 0.69) 7	−2.03 (−2.90, −0.16) 7	0.051
**Depression scale *Z*-score, median (IQR)** Missing, *n*	ND	−0.47 (−1.18, 0.63) 6	−0.32 (−0.83, 0.61) 6	0.60

No estimate was determined if three or fewer values were available, and such cases are shown in the table as ND (not determined). Comparison of numerical data across the three diagnosis groups was performed using the Kruskal–Wallis test. Categorical variables were compared using the chi-square test or Fisher’s exact test, depending on the expected cell frequencies. *P*-values reported were not adjusted for multiple comparisons. An asterisk (*) denotes statistical significance.

Abbreviations: ADEM: acute disseminated encephalomyelitis, CIS: clinically isolated syndrome, EDSS: Expanded Disability Status Scale, IMT: immune modulating therapy, IQR: interquartile range, MOGAD: myelin oligodendrocyte glycoprotein antibody-associated disease, MS: multiple sclerosis, NA: not applicable, ND: not determined, NPA: neuropsychological assessment, ON: optic neuritis, SD: standard deviation, TM: transverse myelitis.

a Other/polyfocal phenotype includes cortical encephalitis, multifocal brain involvement without encephalopathy, or combined clinical phenotypes (e.g. optic neuritis in combination with transverse myelitis).

b Percentages for treatment type are calculated among patients who received any acute treatment; patients may have received more than one acute treatment.

c Percentages are calculated among patients who received maintenance treatment.

### Test results

At the group level, full-scale intelligence quotient (FSIQ) was within normal range across all diagnosis groups, with median values of 104 (IQR: 92–112) for aMOGAD, 97 (IQR: 93–116) for naMOGAD and 98 (IQR: 94–103) for MS children. FSIQ scores did not differ significantly between diagnosis groups (*p* = 0.84). For FSIQ, one child with naMOGAD scored in the very low range (Figure 2).

**Figure 2.**
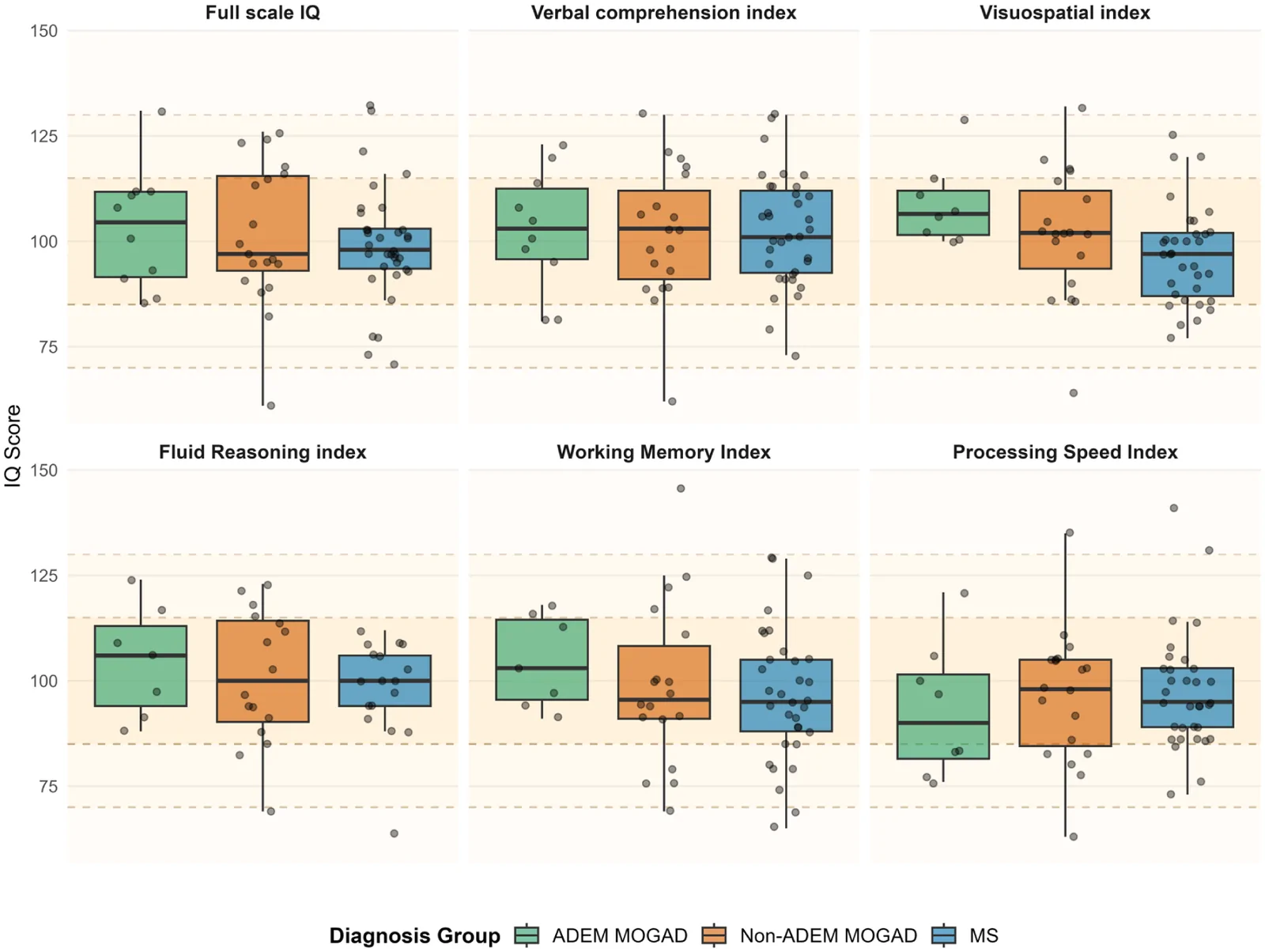
Boxplots indicating full-scale intelligence quotient (IQ) and intelligence subindex scores across all three diagnosis groups. IQ ranges are categorized as follows: below 70 (impaired), 70–85 (below average), 85–115 (average), 115–130 (high average to superior) and above 130 (very superior). Shading is applied using a yellow gradient to represent these categories.

At the group level, children with ADEM did not show statistically significant deviations from normative reference values. In the naMOGAD group, Bourdon-Vos Test (BVT) SD (standard deviation) and BVT Time *Z*-scores were significantly lower compared to reference data, but only BVT time *Z*-score remained significant after FDR correction (BVT SD median *Z*-score: −1.10, *p* = 0.011, adj. *p* = 0.079; BVT time median *Z*-score: −1.48, *p* = 0.001, adj. *p* = 0.020). Children with MS exhibited deficits in multiple tests, including BVT SD (median *Z*-score: −2.90, *p* < 0.001, adj. *p* < 0.001), BVT time (median *Z*-score: −1.86, *p* < 0.001, adj. *p* = 0.001), Rey Complex Figure Test (RCFT) immediate recall (median *Z*-score: −0.67, *p* = 0.001, adj. *p* = 0.005), RCFT delayed recall (median *Z*-score: −0.69, *p* < 0.001, adj. *p* = 0.001), RCFT copy (median *Z*-score: −0.39, *p* = 0.010, adj. *p* = 0.031) and Beery visuomotor integration (Beery VMI, median *Z*-score: −0.73, *p* = 0.014, adj. *p* = 0.035). Figure 3 shows an integrated overview of median *Z*-scores across subtests, organized by cognitive subdomain. In unadjusted analyses comparing MS with the total MOGAD group, differences were observed for BVT SD, RCFT immediate and delayed recall, Visual Spatial Index (VSI), and 15‑Word Test immediate recall (15‑WT Immediate), with lower scores in MS on most measures, whereas 15-WT Immediate score was higher in MS. However, none of these differences remained statistically significant after FDR correction. When restricting the comparison to MS versus naMOGAD, differences were most pronounced for RCFT immediate and delayed recall. All test *Z*-scores and pairwise comparisons are listed in Supplementary 2.

**Figure 3.**
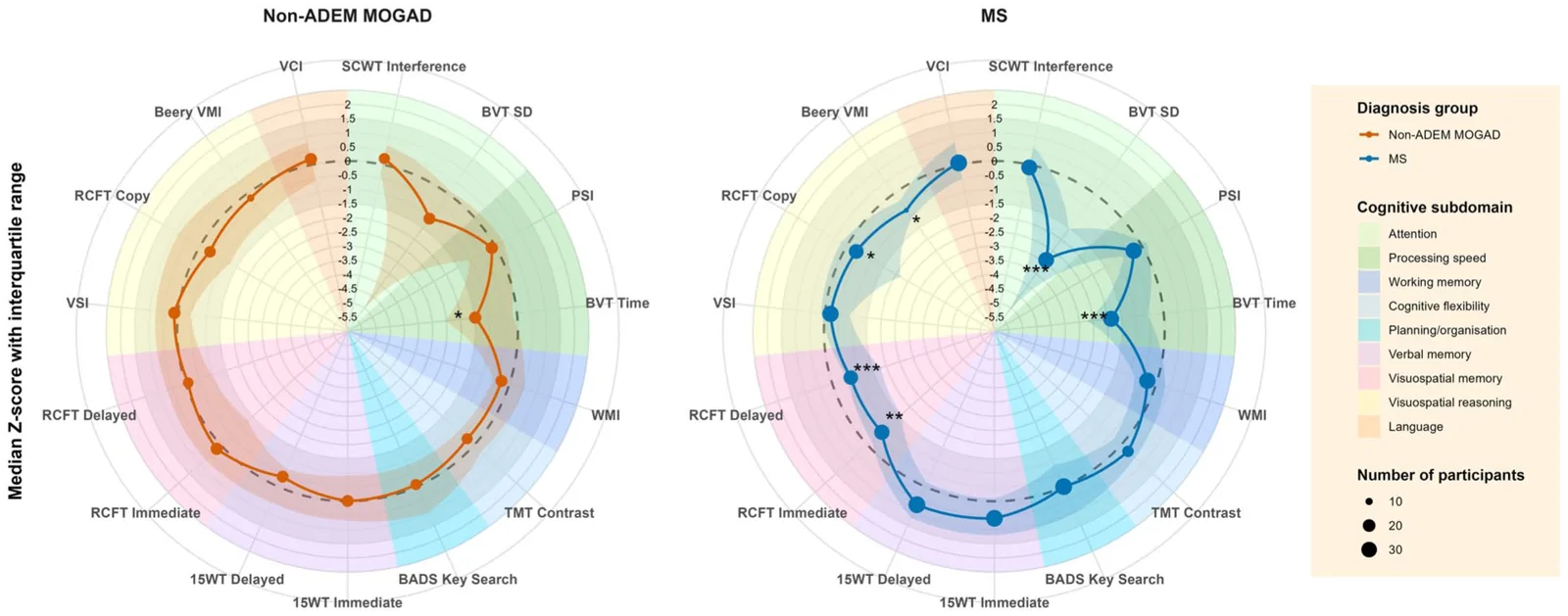
Polar plots represent the median *Z*-scores per test in Non-ADEM MOGAD and MS, with data points sized according to the number of children assessed. Shading reflects the interquartile range. The test score data points are grouped by cognitive subdomain. The dashed line indicates a *Z*-score of 0, with asterisks marking significant deviations from the control group (*Z* = 0), where * denotes adjusted *p* < 0.05, ** denotes adjusted *p* < 0.01 and *** denotes adjusted *p* < 0.001.

### Prevalence of CI

MCI was observed in 47% within the MS group and in a comparable proportion in the naMOGAD group (43%). SCI was most prevalent in the MS group (25%) followed by the naMOGAD group (14%). There were no statistically significant differences between the two groups.

### DSIs

As summarized in Table 3, complex attention was the most affected domain in both MS and naMOGAD children, with significantly lower median composite *Z*-scores compared with normative expectations (MS: −1.24 [IQR: −2.06, −0.60], adj. *p* < 0.001; naMOGAD: −1.12 [IQR: −2.34, 0.11], adj. *p* = 0.019). The proportion of children with DSI (composite *Z*-score < −1.5) in complex attention was significantly higher than expected based on a normally distributed normative reference population (MS: 36.1%, adj. *p* < 0.001; naMOGAD: 38.1%, adj. *p* < 0.001). Children with MS additionally showed a reduced median composite *Z*-score in the visuospatial processing domain (−0.51 [IQR: −1.02, 0.15], adj. *p* = 0.005). Severe DSI was most commonly observed in complex attention (MS: 27.8%, adj. *p* < 0.001; naMOGAD: 33.3%, adj. *p* < 0.001), whereas it was rare in the other domains (not shown in Table 3). In the aMOGAD group, there was no notable DSI.

**Table 3.** Domain analysis across diagnosis groups.

	ADEM MOGAD				Non-ADEM MOGAD				MS			
	Composite *Z*-score, median (IQR)	Adj. *p*-value	Impairment, *n*/*N* (%)	Adj. *p*-value	Composite *Z*-score, median (IQR)	Adj. *p*-value	Impairment, *n*/*N* (%)	Adj.*p*-value	Composite *Z*-score, median (IQR)	Adj.*p*-value	Impairment, *n*/*N* (%)	Adj. *p*-value
Complex attention	−0.20 (−1.13, 0.40)	0.95	1/9 (11.1%)	1.0	−1.12 (−2.34, 0.11)	0.019*	8/21 (38.1%)	<0.001*	−1.24 (−2.06, −0.60)	<0.001*	13/36 (36.1%)	<0.001*
Executive functioning	0.87 (−0.10, 1.13)	0.95	0/7	1.0	−0.18 (−0.76, 0.17)	0.51	0/21	1.0	0.022 (−0.79, 0.67)	0.56	2/36 (5.6%)	1.0
Memory	0.15 (−0.19, 1.10)	0.82	0/5	1.0	−0.001 (−0.52, 0.69)	0.60	1/20 (5.0%)	1.0	0.083 (−0.79, 0.43)	0.56	3/36 (8.3%)	1.0
Visuospatial reasoning	0.00 (−0.14, 0.40)	0.95	1/10 (10.0%)	1.0	0.020 (−0.74, 0.77)	0.60	1/21 (4.8%)	1.0	−0.51 (−1.02, 0.15)	0.005*	5/36 (13.9%)	0.45
Language	0.20 (−0.28, 0.83)	0.95	0/10	1.0	0.20 (−0.60, 0.80)	0.72	1/19 (5.3%)	1.0	0.067 (−0.50, 0.80)	0.86	1/35 (2.9%)	1.0

Domain‑level analyses were performed by comparing composite *Z*‑scores for each of the five principal cognitive domains with a reference value of *Z* = 0, representing the normative population mean derived from age‑adjusted test norms. Based on the a priori hypothesis that performance would be below normative levels, one‑sample Wilcoxon signed‑rank tests with a one‑sided alternative (median *Z* < 0) were applied. False discovery rate correction was applied to account for multiple domain comparisons. In addition, the proportion of participants with a composite *Z*‑score below −1.5, indicating domain‑specific cognitive impairment, was compared with the expected proportion of impaired individuals in a normally distributed reference population (6.7%) using one‑sample binomial tests, with false discovery rate correction for multiple comparisons. An asterisk (*) denotes statistical significance.

Abbreviations: ADEM: acute disseminated encephalomyelitis, IQR: interquartile range, MOGAD: myelin oligodendrocyte glycoprotein antibody-associated disease, MS: multiple sclerosis.

To increase the granularity of the analysis, we examined cognitive deficits in specific subdomains, again distinguishing between mild and severe impairments. At the subdomain level, deficits in attention and processing speed were prominent and often severe for both naMOGAD (attention: 40.0%, *p* < 0.001; processing speed: 38.1%, *p* < 0.001) and MS (attention: 41.7%, *p* < 0.001; processing speed: 31.4%, *p* < 0.001). While memory deficits did not reach statistical significance at the domain level, subdomain analysis revealed that several children with MS exhibited significant visuospatial memory difficulties (25.8%, *p* < 0.01). The heatmap in Figure 4 provides an overview of cognitive deficits across subdomains.

**Figure 4.**
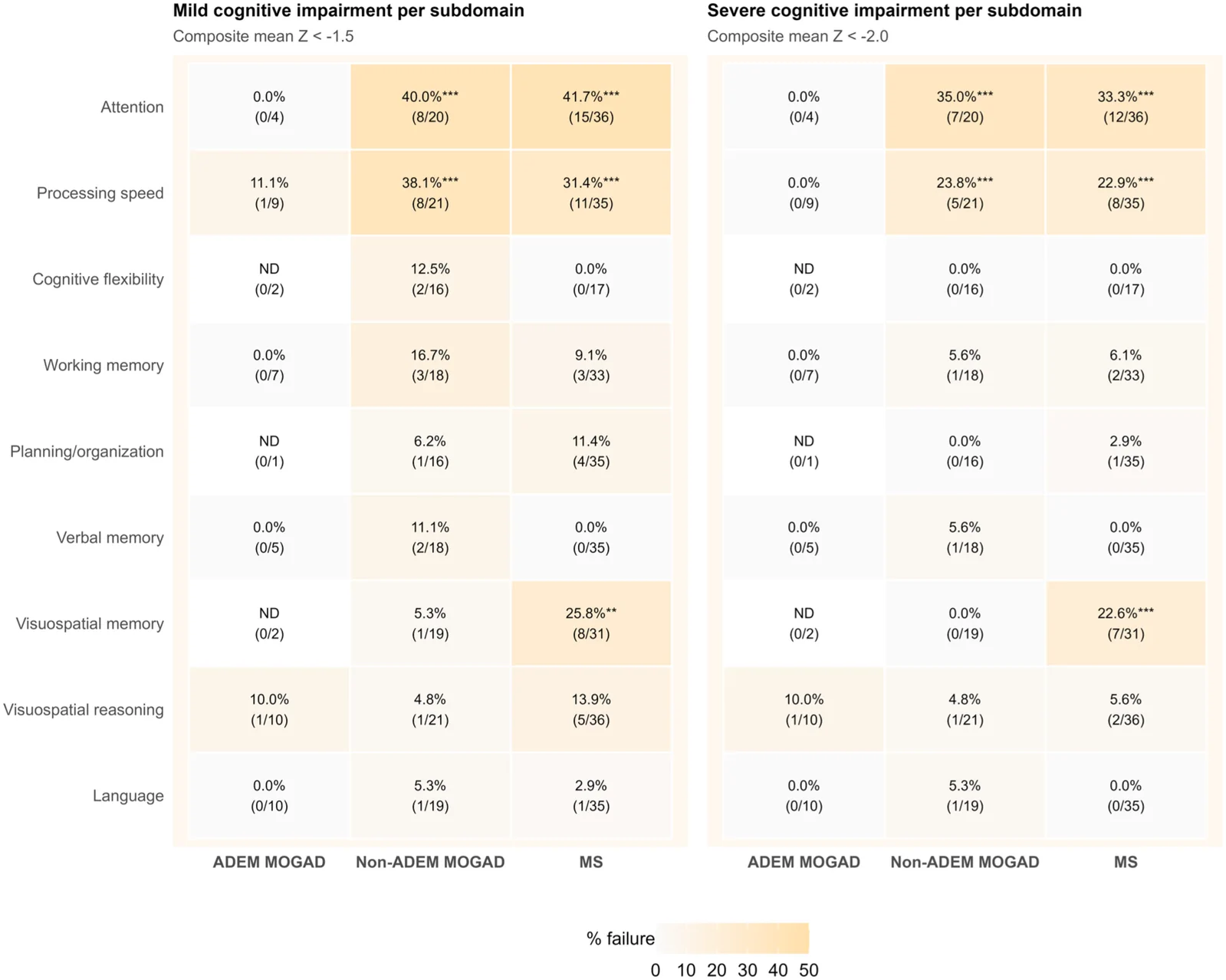
Heatmaps showing the number and percentage of children with composite *Z*-scores below the thresholds of −1.5 (mild impairment) and −2.0 (severe impairment) for each cognitive subdomain. Observed impairment rates were compared with expected proportions in a normative population (6.7% for *Z* < −1.5% and 2.3% for *Z* < −2.0) using one‑sample binomial tests with false discovery rate correction. Asterisks denote adjusted significance levels (*p < 0.05; **p < 0.01; ***p < 0.001). If fewer than three test scores were available, no percentage was calculated and the cell is marked as ND (not determined). Data are presented for all three diagnosis groups.

### Factors associated with CI

Table 4 compares children with naMOGAD and MS based on whether they fulfill criteria for MCI. There were no statistically significant differences in demographical, clinical and radiological factors. In the naMOGAD group, cerebral lesions were present in 6/9 children with CI compared to 5/12 without CI, with cerebral lesions observed across all clinical presentations in the CI group (2/3 polyfocal, 2/3 myelitis, 2/3 optic neuritis). In the unimpaired group, cerebral lesions were present in 0/2 polyfocal, 3/4 myelitis, 1/5 optic neuritis cases, and in the single infratentorial case. Resolving lesions were observed in 5/5 children with CI (*n* = 1 missing) and 4/5 children in the unimpaired group, though complete resolution was observed in only 1/5 children with CI compared to 2/4 without CI. For children with MS, patient and parent-reported fatigue scores were significantly lower in the CI group (patient-reported PedsQL fatigue *Z*-score: −2.61 vs −0.75, *p* = 0.004; parent-reported PedsQL fatigue *Z*-score: −2.58 vs −0.66, *p* = 0.01). When applying a cutoff for clinically significant fatigue (*Z* < −1.5), fatigue was more frequently observed in children with MS, reaching statistical significance for child‑reported fatigue and borderline significance for parent‑reported fatigue. No difference in CDI *Z*-scores was observed.

**Table 4.** Factors associated with cognitive impairment.

	Non-ADEM MOGAD			MS		
	No cognitive impairment *N* = 12	Cognitive impairment *N* = 9	*p*-value	No cognitive impairment *N* = 19	Cognitive impairment *N* = 17	*p*-value
**Demographical factors**						
**Age at time of NPA, mean (SD)**	12.6 (3.0)	13.2 (3.2)	0.80	15.1 (2.4)	15.6 (1.9)	0.63
**Female sex**, *n* **(%)**	6/12 (50.0%)	3/9 (33.3%)	0.66	14/19 (73.7%)	12/17 (70.6%)	1.0
**Born in Netherlands**, *n* **(%)** Missing, *n*	10/11 (90.9%) 1	7/9 (77.8%) 0	0.17	15/18 (83.3%) 1	15/16 (93.8%) 1	0.80
**European origin**, *n* **(%)** Missing, *n*	7/11 (63.6%) 1	5/9 (55.6%) 0	1.0	9/18 (50.0%) 1	8/17 (47.1%) 0	1.0
**Clinical factors**						
**Clinical phenotype**, *n* **(%)**			0.82			0.57
CIS hemispheral	0/12	0/9		2/19 (10.5%)	1/17 (5.9%)	
CIS infratentorial	1/12 (8.3%)	0/9		7/19 (36.8%)	4/17 (23.5%)	
ON	5/12 (41.7%)	3/9 (33.3%)		1/19 (5.3%)	3/17 (17.6%)	
Other/polyfocal^a^	2/12 (16.7%)	3/9 (33.3%)		1/19 (5.3%)	3/17 (17.6%)	
TM	4/12 (33.3%)	3/9 (33.3%)		8/19 (42.1%)	6/17 (35.3%)	
**Disease duration at time of NPA, mean (SD)**	10.2 (4.5)	10.7 (2.6)	0.80	8.7 (3.7)	9.1 (3.4)	0.72
**Relapsing disease**, *n* **(%)**	3/12 (25.0%)	2/9 (22.2%)	1.0	7/19 (36.8%)	6/17 (35.3%)	1.0
**EDSS at time of NPA, median (IQR)**	1.25 (1.0–2.0)	1.5 (1.0–1.5)	0.53	1.5 (1.5–2.0)	2.0 (1.5–2.5)	0.51
**Acute treatment at first event**, *n* **(%)**	12/12 (100%)	9/9 (100%)	NA	9/19 (47.4%)	7/17 (41.2%)	0.97
**Oral corticosteroid taper at first event**, *n* **(%)**	1/12 (8.3%)	1/9 (11.1%)	1.0	2/19 (10.5%)	1/17 (5.9%)	1.0
**IMT maintenance treatment**, *n* **(%)**	1/12 (8.3%)	2/9 (22.2%)	0.55	15/19 (78.9%)	15/17 (88.2%)	0.66
Moderate efficacy therapy^b^	NA	NA		11/15 (73.3%)	11/15 (73.3%)	
High efficacy therapy^b^	NA	NA		4/15 (26.7%)	4/15 (26.7%)	
**Radiological factors**						
**Cerebral involvement on first MRI**, *n* **(%)** Missing, *n*	5/12 (41.7%) 0	6/9 (66.7%) 0	0.39	17/19 (89.5%) 0	17/17 (100%) 0	0.49
**Resolving brain lesions on follow-up MRI**, *n* **(%)**	4/5 (80%)	5/5 (100%)	0.52	NA	NA	NA
Partially resolved^c^	2/4 (50%)	4/5 (80%)		NA	NA	
Completely resolved^c^	2/4 (50%)	1/5 (20%)		NA	NA	
Missing, *n*	0	1				
**⩾** **9 T2/FLAIR lesions on first brain MRI**, *n* **(%)** Missing, *n*	NA	NA	NA	9/17 (52.9%) 2	11/17 (64.7%) 0	0.73
**MRI black holes on first brain MRI**, *n* **(%)** Missing, *n*	NA	NA	NA	6/16 (37.5%) 3	10/16 (62.5%) 1	0.29
**Patient-reported outcomes**						
**PedsQL fatigue *Z*-score (child), median (IQR)** Missing, *n*	−0.69(−1.90, −0.18) 4	−0.91 (−1.46, 0.35) 2	0.82	−0.75 (−1.30, 0.51) 2	−2.61 (−3.10, −1.29) 3	0.005*
**Significant fatigue***^d^* **(child)**, *n* **(%)** Missing, *n*	3/8 (37.5%) 4	2/7 (28.6%) 2	1.0	3/16 (18.8%) 3	10/14 (71.4%) 3	0.009*
**PedsQL fatigue *Z*-score (parent), median (IQR)** Missing, *n*	0.16 (−1.04, 1.05) 3	−0.49 (−1.26, 0.6) 4	0.50	−0.66 (−2.20, 0.57) 2	−2.58 (−3.79, −2.03) 4	0.012*
**Significant fatigue***^d^* **(parent)**, *n* **(%)** Missing, *n*	2/9 (22.2%) 3	1/5 (20.0%) 4	1.0	7/16 (43.8%) 3	11/13 (84.6%) 4	0.052
**CDI *Z*-score, median (IQR)** Missing, *n*	−0.77 (−1.15, 0.09) 4	0.33 (−0.87, 0.63) 2	0.82	−0.32 (−1.10, 0.65) 2	−0.25 (−0.47, 0.61) 4	0.48

For the Non-ADEM MOGAD and MS subgroups, children with cognitive impairment versus those without cognitive impairment were compared, based on the definition of MCI. Statistical differences were assessed using the Kruskal–Wallis test or chi-square/Fisher’s exact test, as appropriate. *P*-values were not adjusted for multiple comparisons. An asterisk (*) denotes statistical significance.

Abbreviations: CDI: Children’s Depression Inventory, CIS: clinically isolated syndrome, EDSS: Expanded Disability Status Scale, FLAIR: fluid-attenuated inversion recovery, IMT: immunomodulating therapy, IQR: interquartile range, MOGAD: myelin oligodendrocyte glycoprotein antibody-associated disease, NA: not applicable, NPA: neuropsychological assessment, ON: optic neuritis, PedsQL: Pediatric Quality of Life Inventory, TM: transverse myelitis.

a Other/polyfocal phenotype includes cortical encephalitis, multifocal brain involvement without encephalopathy, or combined clinical phenotypes (e.g. optic neuritis in combination with transverse myelitis).

b Percentages are calculated among patients who received maintenance treatment.

c Percentages are calculated among patients with resolving brain lesions on MRI.

d Significant fatigue was defined as a PedsQL Fatigue *Z*-score below −1.5.

### Discrepancy between objective CI and self-reported cognitive complaints

Cognitive complaints were reported by 53% of children with MS. Among children with aMOGAD, approximately one-third reported such complaints, whereas 29% of those with naMOGAD reported cognitive problems. Cohen’s kappa was 0.34 (95% CI: 0.03 to 0.63) for the MS group, indicating moderate agreement and −0.12 (95% CI: −0.51 to 0.28) for the naMOGAD group, indicating no agreement between cognitive complaints and objective CI.

## Discussion

This study provides a comprehensive evaluation of cognitive functioning in cADS using an extensive neuropsychological test battery covering all principal cognitive domains. A substantial proportion of children fulfilled criteria for CI, with 47% in MS and 43% in naMOGAD, despite overall intelligence remaining within normal range. MS children performed significantly worse on the BVT SD, BVT Time, RCFT tasks and Beery VMI. Children with naMOGAD showed similar deficits on the BVT, particularly BVT Time. Complex attention was most affected across MS and naMOGAD, with MS children also exhibiting visuospatial deficits. Notably, there was limited agreement between self-reported cognitive complaints and objective test results.

We found a higher rate of CI compared to previous studies in pediatric MS.^3^ A key difference in our approach was the use of robust domain-dependent criteria, requiring deficits in at least two domains to classify impairment, which enhances specificity and aligns with contemporary neuropsychological practice. We used a *Z*-score threshold of <–1.5 to define mild CI, as this cutoff is commonly regarded as clinically meaningful, and severe CI by additionally applying a strict cutoff of <–2.0.^15,16^ Using this latter threshold, as expected, we observed a lower CI prevalence of 25% in the MS subgroup. Another difference with previous studies is that we used a comprehensive test battery covering all principal cognitive domains as defined by *DSM*-5.^14^ Moreover, we included sensitive complex attention measures which represent a neurobiologically plausible area of vulnerability in MS.^17^ Previous research has shown that the risk of detecting CI increases with the number of tests performed.^18^ Together these methodological choices likely account for the higher CI prevalence observed in our cohort.

Within our MOGAD cohort, we observed a high prevalence of MCI in the non-ADEM subgroup, comparable to children with MS. Although no formal estimates of CI prevalence have been reported in children with MOGAD, studies in adults indicate rates between 11% and 36%.^5–7^ Pediatric data are very limited, with only a small number of studies, typically involving small samples sizes, restricted cognitive assessments, and focusing primarily on ADEM. Despite this heterogeneity, the available literature consistently notes that some children show difficulties on tasks mainly concerning processing speed, memory and working memory.^8^ Although the test battery was comprehensive, the young age of aMOGAD participants limited the assessments, hindering a deeper analysis of CI in the aMOGAD subgroup.

The neurocognitive profile of MS children was characterized predominantly by reduced sustained attention and processing speed and deficits in visuospatial reasoning and visuospatial memory. In contrast, language and executive functions were preserved. These observations are broadly consistent with previous reports in pediatric MS.^1^ Although language impairments have been previously reported, they may not have been captured in our study due to limited language testing. In naMOGAD, complex attention emerged as the only DSI, yet deficits were common and often severe.

From a clinical standpoint, the high prevalence of CI across the cADS spectrum underscores the need for targeted cognitive monitoring, especially given the limited concordance between self‑reported complaints and objective test results. The heterogeneity of pediatric MS necessitates comprehensive testing over screening to capture domain-specific deficits and ensure findings are properly contextualized in order to inform individualized clinical management. In settings where a full NPA is unfeasible due to resource constraints, screening instruments such as the Brief International Cognitive Assessment for Multiple Sclerosis^19^ (BICAMS) and the Cogstate Brief Battery^20^ (CBB) may provide a pragmatic alternative. Our findings suggest that these tools are sensitive to the domains most frequently affected in pediatric MS, such as complex attention and visuospatial memory. However, to improve diagnostic yield, future pediatric screening instruments should prioritize enhanced sensitivity to visuospatial processing deficits. Conversely, screening could be more focused in naMOGAD, specifically targeting complex attention and processing speed.

From a neurobiological perspective, the cognitive profile we observed in pediatric MS likely reflects the presence of multifocal white matter demyelination or diffuse CNS inflammation and consequent network inefficiency.^21,22^ Thalamic pathology may further mediate these deficits, as smaller thalamic volumes and thalamic dysconnectivity have been associated with worse cognitive performance in both adult^23,24^ and pediatric MS^25^ cohorts. As attentional deficits and reduced processing speed represent the most prominent cognitive deficits in MOGAD as well, this further points toward an increased vulnerability of large‑scale brain networks to inflammatory injury. A recent study indicated that MOGAD patients with optic neuritis experience significant disruptions in white matter networks, supporting this hypothesis.^26^ These network‑level disturbances may arise from microstructural white‑matter damage or distant effects of focal lesions with trans-synaptic degeneration as a possible underlying mechanism,^27^ or a combination. We found that fatigue was significantly associated with CI, raising the question whether fatigue negatively influences test performance, cognitive deficits lead to fatigue, or both represent parallel manifestations of a shared underlying brain network insufficiency.^28–30^ No other demographic, clinical, or radiological measures were significantly associated with CI. Although the modest sample size limits detection of subtle associations, this finding suggests that traditional markers do not capture early network‑level dysfunction. Exploratory observations may, however, point to a possible role for cerebral lesion involvement in cognitive outcome in naMOGAD. Cerebral lesions were more frequently observed in children with CI than without, and complete lesion resolution appeared somewhat less frequent in the CI group.

Interestingly, visuospatial processing and visual memory difficulties were observed only in MS and not in naMOGAD. This is consistent with an adult comparative study reporting poorer visual memory performance in MS despite otherwise similar cognitive profiles.^31^ This difference is unlikely to be explained solely by optic neuritis, as children in the naMOGAD group frequently experienced optic neuritis. Rather, it may reflect disruption of higher‑order cortical visual networks, indicating a contribution of focal involvement of specific visual pathways and cortical regions in pediatric MS. Accordingly, visuospatial performance may serve as a functional correlate of visual network integrity, bridging sensory input and cognitive processing, and may complement structural assessments of the visual system, such as optical coherence tomography and visual evoked potentials in pediatric MS.

Several methodological limitations should be considered when interpreting our findings. Test selection and categorization strongly shape domain-level outcomes. In our study, the BVT heavily impacted both attention and processing speed findings and consequently CI prevalence. Its high impairment rates, contrasting with the relatively unaffected Stroop Color Word Test and processing speed index scores, likely stem from its sustained attentional demands. Nonetheless, this discrepancy raises the question of whether the BVT detects subtle attentional and information processing inefficiencies missed by less sensitive and broader indices, or whether it overestimates deficits due to task-specific or outdated norms. The absence of a healthy control group is relevant as certain cognitive functions, particularly attention, may have shifted over recent decades due to societal changes, although no empirical data exist. In addition, the RCFT illustrates how a single test affects multiple domains: its recall performance partly reflects the quality of the initial copy, blurring distinctions between visuospatial reasoning and memory. To mitigate these concerns, we used composite *Z*-scores for more robust domain and subdomain analysis. Furthermore, not all participants completed the full neuropsychological protocol due to time, logistics or young age.

## Conclusion

Our findings demonstrate that cognitive difficulties, particularly in complex attention, are prevalent in pediatric MS and naMOGAD. In the MS group, visuospatial abilities appear to be an additional vulnerable area. The neurocognitive profile we outlined offers practical guidance for targeted cognitive monitoring and interventions and helps select relevant measures for further research. An important consideration remains whether the frequent attentional weaknesses reflect disease-specific mechanisms or broader societal trends. Regardless, our findings underscore the importance of early, systematic cognitive assessment in cADS in clinical settings.

## Supplemental Material

sj-docx-1-msj-10.1177_13524585261465586 – Supplemental material for Frequent early cognitive impairment in both pediatric MS and MOGAD Supplemental material, sj-docx-1-msj-10.1177_13524585261465586 for Frequent early cognitive impairment in both pediatric MS and MOGAD by Michiel Simon Jan Buijze, Sandy Molenaar, Rosa Verkerk, Maud Alida Quist, Willemijn Marije Carolien van der Vlugt and Rinze Frederik Neuteboom in Multiple Sclerosis Journal

## Data Availability

The datasets generated during and/or analyzed during the current study are available from the corresponding author on reasonable request.
