# Supplementary material for: Frequent early cognitive impairment in both pediatric MS and MOGAD
Source: Mult Scler. 2026 Aug 6;32(11):1168–81. doi: 10.1177/13524585261465586 (PMC13646429; doi:10.1177/13524585261465586)
Supplement: Supplementary file 1 [file sj-docx-1-msj-10.1177_13524585261465586.docx]

**Supplementary 1 - Neuropsychological assessment protocol**

**Procedure**

Prior to the neuropsychological assessment, parents or legal guardians and patients aged 12 years and older were provided with standardized written information outlining the aims and procedures. Assessments were carried out by a trained neuropsychologist in a dedicated testing room at Erasmus Medical Centre – Sophia Children’s Hospital. Each NPA began with the administration of the age‑appropriate Wechsler Intelligence Scale. After a mandatory pause of at least ten minutes, the remaining tests were administered in a predetermined sequence. Additional rest periods were offered whenever deemed necessary or upon request. The full assessment was completed within a maximum duration of approximately three hours. In most cases, the NPA was finalized within a single session. However, when a participant could not sustain the full session, the assessment was divided across two separate days.

**Framework for cognitive domain classification**

The cognitive test battery was structured according to the framework established by the Diagnostic and Statistical Manual of Mental Disorders, 5^th^ edition (DSM-5) criteria for neurocognitive disorders. The six principal cognitive domains are: 1) Complex attention, 2) Executive function, 3) Learning and memory, 4) Perceptual-motor function, 5) Language and 6) Social cognition. Social cognition was excluded due to methodological considerations. To obtain a more fine-grained profile, three domains were subdivided: complex attention into attention and processing speed; executive function into planning/organization, cognitive flexibility and working memory; and learning and memory into verbal and visuospatial components. This DSM-5 approach enables comprehensive and standardized profiling of cognitive functioning, identifying precise patterns of cognitive decline, improving comparability across studies, and helping to relate functional impairments more accurately to underlying neuropathology.

Based on this framework, we selected tests for each domain and subdomain according to their psychometric properties, clinical relevance and availability of age-appropriate normative data. The selected tests and their mapping onto the DSM-5 domains are summarized in the table below.

| Included measures per cognitive domain | | | |
| --- | --- | --- | --- |
| **Cognitive domain** | **Test** | **Measure(s)** | **Abbreviation(s)** |
| Complex attention | BVT | Standard deviation score  Mean time score | BVT SD  BVT Time |
|  | SCWT | Interference score | SCWT Interference |
|  | Age 6-16: WISC-V-NL  Age 16+: WAIS-IV-NL | Processing Speed index | PSI |
| Executive function | Age 6-16: BADS-C  Age 16+: BADS | Key Search subtest score | BADS Key Search |
|  | DKEFS TMT | Contrast score | TMT Contrast |
|  | Age 6-16: WISC-V-NL  Age 16+: WAIS-IV-NL | Working Memory Index | WMI |
| Memory | Age 6-14: 15-WT-C  Age 14+: 15-WT-A | Immediate recall and delayed recall scores | 15-WT Immediate  15-WT Delayed |
|  | Age 5-15: ROFC  Age 15+: RCFT | Immediate recall and delayed recall scores | RCFT Immediate  RCFT Delayed |
| Visuospatial cognition | Age 5-15: ROFC  Age 15+: RCFT | Copy trial score | RCFT Copy |
|  | Age 6-16: WISC-V-NL  Age 16+: WAIS-IV-NL | Visuospatial index  Visuospatial index (adapted construct) | VSI |
|  | Beery BDT | Visual motor integration score | Beery VMI |
| Verbal abilities | Age 6-16: WISC-V-NL  Age 16 and over: WAIS-IV-NL | Verbal comprehension index | VCI |
| *Abbreviations: 15-WT-A: 15-Words Test version A; 15-WT-C: 15-Words Test for Children; Beery BDT: Beery Buktenica Developmental Test; BADS: Behavioural Assessment of the Dysexecutive Syndrome; BADS-C: Behavioural Assessment of the Dysexecutive Syndrome for Children; BVT: Bourdon-Vos Test; D-KEFS TMT: Delis-Kaplan Executive Function System Trail Making Test; EF: executive functioning; PSI: Processing Speed Index; RCFT: Rey Complex Figure Test; ROCF: Rey-Osterrieth Complex Figure test; SCWT (Netherlands): Stroop Color and Word Test; VCI: Verbal Comprehension Index; VSI: Visual Spatial Index; WAIS-IV-NL: Wechsler Adult Intelligence Scale 4^th^ edition Netherlands; WISC-V-NL: Wechsler Intelligence Scale for Children 5^th^ edition Netherlands; WMI: Working Memory Index.* | | | |

**Wechsler Intelligence Scales**

To evaluate general intellectual ability, we administered the Dutch adaptations of the Wechsler Intelligence Scales. Children and adolescents between 6 and 16 years completed the Wechsler Intelligence Scale for Children, Fifth Edition (WISC‑V‑NL) (Wechsler, 2008/2012). Individuals aged 16 years and older were assessed with the Wechsler Adult Intelligence Scale, Fourth Edition (WAIS‑IV‑NL) (Wechsler, 2014/2017). The Full Scale Intelligence Quotient (FSIQ) was calculated as the sum of the scaled scores of the seven primary subtests: Similarities and Information, Block Design, Matrix Reasoning and Figure Weights, Digit Span, and Symbol Search. In addition, multiple primary index scores were calculated, representing different aspects of intellectual functioning:

- The Processing Speed Index (PSI) is a measure of the speed of cognitive processes and response output.
- The Working Memory Index (WMI) reflects the ability to hold and manipulate information in the mind.
- Visuospatial cognition was assessed using the Block Design and Visual Puzzles subtests from the WISC‑V‑NL and WAIS‑IV‑NL. Within the WISC‑V‑NL, these two tasks together constitute the Visual Spatial Index (VSI). Although the WAIS‑IV‑NL includes the same subtests, they contribute to the Perceptual Reasoning Index rather than a dedicated visuospatial index. To enable direct comparison across the child and adult test batteries, an adapted score based on Block Design and Visual Puzzles was computed for the WAIS‑IV‑NL, mirroring the VSI structure of the WISC‑V‑NL. The VSI reflects non-verbal reasoning skills involved in visual pattern recognition, spatial orientation, and the integration of visual information for problem-solving.
- The Verbal Comprehension Index (VCI) measures the ability to understand, use and reason with words.

**Additional tests**

*Bourdon-Vos Test*

The Bourdon–Vos test was administered to evaluate complex attention. Both the mean time required to complete each row, indexing processing speed, and the standard deviation of row times, reflecting sustained attention, were included in the analyses. Both metrics were converted into age‑adjusted Z‑scores based on normative data derived from a Dutch reference population (Vos, 1988, 1998).

*Stroop Color and Word Test*

The interference score of the Dutch version of the Stroop Color and Word Test (SCWT) was used (Stroop, 1935; Hammes, 1971/1978) to assess attention. Although also considered a measure of inhibition, it is widely used as a measure of selective attention (Nasiri et al., 2023; Wright, 2016). This test can be used for patients aged 8-65. Z‑scores were calculated using normative data adjusted for age, sex, and education, derived from a Dutch reference population (Hammes, 1978; Schmand et al., 2002).

*Rey Complex Figure Test/ Rey-Osterrieth Complex Figure test*

Visuospatial cognition and visual memory were assessed using the Rey–Osterrieth Complex Figure (ROCF) in participants aged 5 to 15 years (Bernstein & Waber, 1996), and the Rey Complex Figure Test (RCFT) in individuals aged 15 years and above (Meyers & Meyers, 1995). The evaluation included performance on the copy trial, as well as the immediate and delayed recall trials. Raw scores from these components were converted to Z‑scores based on age‑adjusted normative data, derived from a United States reference population (Bernstein & Waber, 1996; Meyers & Meyers, 1995). No language or cultural adaptations were required for the Rey–Osterrieth Complex Figure Test, as it is non‑verbal and probably independent of cultural influences.

*15-Words Test*

Verbal memory was evaluated using the 15-Words Test for Children (15-WT-C) for patients aged 6-14 (Kingma & Van den Burg, 2008). This is the Dutch version of Rey’s Auditory-Verbal Learning Test (RAVLT) (Rey, 1964). For individuals aged 14 years and above, version A of the adult 15-Words Test (15‑WT‑A) was administered (Saan & Deelman, 1986). Z-scores were calculated based on age, gender, and education adjusted norms from a Dutch reference group.

*Behavioural Assessment of the Dysexecutive Syndrome*

Executive functioning was evaluated using the Dutch versions of the Behavioural Assessment of the Dysexecutive Syndrome (BADS). Participants between 6 and 16 years completed the BADS‑C (Wilson, 1996/2000), whereas individuals aged 16 and above were assessed with the adult BADS (Emslie, 2003/2006). For the purposes of this study, analyses focused exclusively on the Key Search task, which is intended to capture the ability to plan in a structured and goal‑directed manner. Raw performance scores from both test versions were transformed into Z‑scores using normative data adjusted for age and intellectual level (Wilson, 1996/2000; Kalff, 1999). Dutch normative data were available only for the adult BADS version.

*D-KEFS Trail Making Test*

To assess executive functioning, the Dutch version of the Delis-Kaplan Executive Function System Trail Making Test (D-KEFS TMT) was used (Delis et al., 2001), which is standardized for individuals aged 8-89 years. For this study, only the contrast score between the fourth condition and conditions two and three was analysed as a metric for cognitive flexibility. Age-based normative data derived from a United Sates reference population from the manual were used to compute Z-scores (Delis et al., 2001).

*Beery Buktenica Developmental Test*

The Beery-Buktenica Development Test was administered as a measure of visuospatial cognition (Beery & Beery, 2010). The test is suitable for individuals aged 2-99 years. In this study, only the visual-motor integration subtest was used, regarded as a robust indicator of visuomotor construction skills. Z-scores were calculated using age-based normative data derived from a United States reference population, as provided in the test manual (Beery & Beery, 2010).

**Questionnaires**

*PedsQL Fatigue*

Fatigue was assessed using the Dutch version of the Pediatric Quality of Life Inventory Multidimensional Fatigue Scale (Gordijn, 2011). This 18-item instrument measures general fatigue, sleep fatigue and cognitive fatigue by rating how often a particular problem occurred in the past month. Higher scores indicate fewer symptoms of fatigue. Raw scores can be converted to Z-scores based on age-based reference norms. It comprises parallel child self-reports for various age groups en parent proxy-reports.

*CDI*

Depressive symptoms were assessed using the Dutch version of the Children’s Depression Inventory (Roelofs 2010). The CDI is a 27-item self-report questionnaire in which respondents choose one of three statements per item that best describes how they have felt over the last two weeks. Each item is scored from 0 to 2, yielding a total score ranging from 0 to 54, with higher scores indicating greater depressive symptomatology. Raw scores can be converted to standardized z‑scores based on normative data.

**Supplementary 2 – Z-scores of test scores for all diagnosis groups**

| **Test scores per cognitive domain, stratified by diagnosis group** | | | | | | | | | | | |
| --- | --- | --- | --- | --- | --- | --- | --- | --- | --- | --- | --- |
|  | **Total MOGAD**  **N = 31** | | | **ADEM MOGAD**  **N = 10** | | **Non-ADEM MOGAD**  **N = 21** | | **MS**  **N = 36** | | **Total MOGAD**  **vs. MS** | **Non-ADEM MOGAD vs. MS** |
|  | **Z-score**  **median (IQR)** | **p-value**  **(adj. p-value)** | | **Z-score**  **median (IQR)** | **p-value**  **(adj. p-value)** | **Z-score**  **median (IQR)** | **p-value**  **(adj. p-value)** | **Z-score**  **median (IQR)** | **p-value**  **(adj. p-value)** | **P-value**  **(adj. p-value)** | **P-value**  **(adj. p-value)** |
| **Complex attention** | | | | | | | | | | | |
| BVT Time | -1.20  (-2.39 – 0.04)  n = 23 | *0.003**  *(0.049)* | | 0.03  (-0.29 – 0.70)  n = 4 | *0.71*  *(0.96)* | -1.48  (-2.55 – -0.18)  n = 19 | *0.001**  *(0.020)* | -1.86  (-2.71 – -0.58)  n = 32 | *<0.001**  *(0.001)* | *0.19*  *(0.47)* | *0.53*  *(0.79)* |
| BVT SD | -1.10  (-4.07 – 0.12)  n = 22 | *0.008**  *(0.070)* | | -1.00  (-1.31 – 0.08)  n = 4 | *0.43*  *(0.96)* | -1.10  (-5.08 – 0.12)  n = 18 | *0.011**  *(0.079)* | -2.90  (-5.20 – -1.55)  n = 31 | *<0.001**  *(<0.001)* | *0.049**  *(0.15)* | *0.17*  *(0.42)* |
| PSI | -0.13  (-1.13 – 0.33)  n = 27 | *0.095*  *(0.36)* | | -0.67  (-1.23 – 0.10)  n = 8 | *0.14*  *(0.45)* | -0.13  (-1.03 – 0.33)  n = 19 | *0.22*  *(0.48)* | -0.33  (-0.73 – 0.20)  n = 33 | *0.039**  *(0.074)* | *0.83*  *(0.92)* | *0.83*  *(0.93)* |
| SCWT Interference | 0.00  (-1.08 – 0.6)  n = 19 | *0.25*  *(0.49)* | | -1.70  (-1.85 – -1.45)  n = 3 | *0.091*  *(0.45)* | 0.22  (-0.38 – 0.70)  n = 16 | *0.69*  *(0.77)* | -0.10  (-0.55 – 0.65)  n = 31 | *0.49*  *(0.67)* | *0.52*  *(0.69)* | *0.78*  *(0.99)* |
| **Executive functioning** | | | | | | | | | | | |
| BADS Key search | -0.33  (-0.67 – 1.00)  n = 17 | | 0.43  (0.64) | ND | *NA* | -0.08  (-0.83 – 1.00)  n = 16 | *0.47*  *(0.70)* | 0.00  (-0.84 – 1.00)  n = 35 | *0.44*  *(0.66)* | *0.86*  *(0.92)* | *0.82*  *(0.93)* |
| TMT Contrast | -0.33  (-0.92 – 0.33)  n = 18 | | 0.23  (0.49) | ND | *NA* | -0.33  (-0.75 – 0.33)  n = 16 | *0.17*  *(0.48)* | 0.33  (-0.33 – 0.67)  n = 17 | *0.67*  *(0.84)* | *0.37*  *(0.61)* | *0.31*  *(0.61)* |
| WMI | -0.20  (-0.60 – 0.87)  n = 25 | | 0.50  (0.68) | 0.20  (-0.30 – 0.97)  n = 7 | *0.85*  *(0.96)* | -0.30  (-0.60 – 0.55)  n = 18 | *0.36*  *(0.59)* | -0.33  (-0.80 – 0.33)  n = 33 | *0.12*  *(0.20)* | *0.51*  *(0.69)* | *0.93*  *(0.93)* |
| **Learning and memory** | | | | | | | | | | | |
| 15-WT Immediate | -0.39  (-0.64 – 0.67)  n = 23 | | *0.41*  *(0.64)* | -0.13  (-0.39 – 0.67)  n = 5 | *0.71*  *(0.96)* | -0.39  (-0.70 – 0.61)  n = 18 | *0.31*  *(0.58)* | 0.70  (-0.15 – 1.20)  n = 35 | *0.98*  *(0.98)* | *0.040**  *(0.15)* | *0.049**  *(0.24)* |
| 15-WT Delayed | -0.10  (-0.95 – 0.55)  n = 23 | | *0.26*  *(0.49)* | 0.39  (0.39 – 0.67)  n = 5 | *0.86*  *(0.96)* | -0.21  (-0.98 – 0.43)  n = 18 | *0.10*  *(0.48)* | 0.29  (-0.20 – 0.70)  n = 34 | *0.88*  *(0.96)* | *0.24*  *(0.48)* | *0.11*  *(0.32)* |
| RCFT Immediate | 0.20  (-1.28 – 0.8)  n = 21 | | 0.76  (0.87) | ND | *NA* | 0.20  (-1.28 – 0. 76)  n = 19 | *0.64*  *(0.77)* | -0.67  (-1.60 – -0.20)  n = 29 | *0.001**  *(0.005)* | *0.020**  *(0.10)* | *0.044**  *(0.24)* |
| RCFT Delayed | 0.50  (-0.67 – 0.71)  n = 17 | | 0.75  (0.87) | ND | *NA* | -0.10  (-0.98 –0.69)  n = 15 | *0.55*  *(0.74)* | -0.69  (-1.80 – -0.60)  n = 26 | *<0.001**  *(0.001)* | *0.006**  *(0.090)* | *0.020**  *(0.24)* |
| **Visuospatial abilities** | | | | | | | | | | | |
| VSI | 0.13  (0.00 – 0.83)  n = 27 | | 0.96  (0.96) | 0.43  (0.10 – 0.80)  n = 8 | *0.99*  *(0.99)* | 0.13  (-0.43 – 0.80)  n = 19 | *0.80*  *(0.80)* | -0.20  (-0.87 – 0.13)  n = 33 | 0.040*  (0.074) | *0.012**  *(0.090)* | *0.10*  *(0.32)* |
| RCFT Copy | -0.48  (-1.17 – 0.72)  n = 18 | | 0.14  (0.41) | ND | *NA* | -0.41  (-1.23 – 0.87)  n = 17 | *0.20*  *(0.48)* | -0.39  (-2.18 – 0.24)  n = 27 | *0.010**  *(0.031)* | *0.55*  *(0.69)* | *0.48*  *(0.79)* |
| BEERY VMI | -0.33  (-0.97 – 0.17)  n = 15 | | 0.070  (0.35) | -0.33  (-0.60 – 0.00)  n = 5 | *0.10*  *(0.45)* | -0.20  (-1.08 – 0.52)  n = 10 | *0.18*  *(0.48)* | -0.73  (-1.07 – -0.33)  n = 9 | *0.014**  *(0.035)* | *0.26*  *(0.48)* | *0.33*  *(0.61)* |
| **Language** | | | | | | | | | | | |
| VCI | 0.2  (-0.47 – 0.93)  n = 29 | | 0.81  (0.87) | 0.20  (-0.28 – 0.83)  n = 10 | *0.78*  *(0.96)* | 0.20  (-0.6 – 0.8)  n = 19 | *0.72*  *(0.77)* | 0.07  (-0.50 – 0.80)  n = 35 | *0.86*  *(0.94)* | *1.0* | *1.0* |
| Median Z-scores including interquartile ranges are displayed for each test score. The one-sample Wilcoxon Signed-Rank Test was used to evaluate whether scores were significantly below normative values (Z < 0). For each test, the number of test scores included in the analysis for each diagnosis group is provided. Additionally, using the two-sample Wilcoxon Signed-Rank Test, the total MOGAD group and the non-ADEM MOGAD group are compared with the MS group to assess potential statistically significant differences between these two groups. The ADEM MOGAD group was excluded from pairwise comparisons due to small sample sizes and substantial missing data. P-values are reported alongside FDR-corrected p-values (in parentheses). An asterisk (*) denotes statistical significance (<0.05). | | | | | | | | | | | |
